# How much survival benefit is necessary for breast cancer patients to opt for adjuvant chemotherapy? Results from a Chilean survey

**DOI:** 10.3332/ecancer.2014.391

**Published:** 2014-01-28

**Authors:** Francisco Acevedo, Cesar Sanchez, Jaime Jans, Solange Rivera, Mauricio Camus, Pelayo Besa

**Affiliations:** 1 Department of Haematology-Oncology, School of Medicine, Pontificia Universidad Catolica de Chile, Santiago 8330032, Chile; 2 Department of Surgery, School of Medicine, Pontificia Universidad Catolica de Chile, Santiago 8330032, Chile; 3 Department of Family and Community Medicine, School of Medicine, Pontificia Universidad Catolica de Chile, Santiago 8330032, Chile; 4 Radiotherapy Service, Clinica Las Condes, Santiago 7591046, Chile

**Keywords:** breast cancer, adjuvant, chemotherapy

## Abstract

**Background:**

Breast cancer (BC) is the leading cause of cancer death in Chilean women. Adjuvant chemotherapy decreases recurrence and death from BC. The recommendation to indicate chemotherapy is complex. Adjuvant! Online is a valuable computational tool to predict survival benefit obtained with adjuvant systemic therapy. Previous studies in Caucasian patients with BC showed that they are willing to receive chemotherapy for a small benefit. No studies, to our knowledge, have been done in the Hispanic or Latino populations.

**Methods:**

We interviewed females with BC who had previously received adjuvant chemotherapy. Age, stage at presentation, time since last chemotherapy, type of chemotherapy, marital status, number of children, and level of education were recorded. We used the graphic representation from Adjuvant! Online to question each patient on how much survival benefit she required to accept chemotherapy.

**Results:**

There were 101 women surveyed. The average age was 55.9 (±10.2), 54.5% had involved lymph nodes, 59.4% were married, and 15.8% did not have parity; 62.3% of females accepted chemotherapy for an absolute survival benefit of 1% or less. In a multivariate analysis, younger (*p* = 0.02) and less-educated patients (*p* = 0.018) were associated with lower survival benefit required to opt for chemotherapy.

**Conclusion:**

In our study, the acceptance of chemotherapy by the Hispanic population requires minimal survival benefit and is in agreement with the Caucasian population reported elsewhere. To our knowledge, our report is the first study that evaluates the perception of Latino patients regarding the benefit of chemotherapy in early BC.

## Introduction

Breast cancer (BC) is the leading cause of cancer death in Chilean women (www.deis.cl). Adjuvant chemotherapy has demonstrated an increase in survival, and also produces adverse effects that can impair quality of life (QoL) or induce morbidity and mortality [1]. In this scenario, the recommendation to use cytotoxic therapy is complex and depends on the evaluation of treatment benefits, the risk for relapse and death, the condition of the patient’s health, and the potential side effects due to treatment.

Most of the prognostic factors that allow us to predict BC relapse are based on the assessment of pathologic tumour features. Tumour size, axillary lymph node involvement, histological grade, presence or absences of hormone receptor status, and epidermal growth factor receptor type 2 (Her-2) are classical determinants to define prognosis. Recently, subgroups of BC based on molecular intrinsic subtypes have been described, with therapeutic and prognostic implications [2]. However, the implementation of these genetic profiles in clinical practice has technical and economical limitations, especially in our country. Therefore, classic pathological elements are still used in the decision making. Nevertheless, the subjective evaluation of these factors does not allow a quantitative assessment of the benefit.

To predict a more accurate prognosis of BC patients and quantitatively estimate the benefit of adjuvant systemic therapy, several combinations of these clinical and/or pathological factors have been used to build different indexes.

Ravdin *et al* [3] built an online application (Adjuvant! Online [www.adjuvantonline.com]) that is able to estimate the probability of relapse and mortality at ten years in an individual patient and has been validated in the Caucasian population and also used in Latin America or the Hispanic population living in the United States [4].

The estimation is calculated considering the age of the patient at diagnosis, comorbidities, presence of oestrogen receptor, tumour size, histological grade, and nodal involvement. In addition to anticipate prognosis, the program permits the estimation of the benefit of different therapeutic options in an easy-to-understand format, adapted to shared-decision making with the patient.

To our knowledge, no data exist in our country or for a Latin population, describing how patients evaluate or analyse the decision to receive chemotherapy, or the magnitude of the benefit required to accept it, when asked in their native language and in their own country.

The aim of this study is to determine the absolute survival benefits that our patients judge adequate to receive chemotherapy and the relation with potential demographic and clinical variables associated with this decision. We decided to use graphics models from Adjuvant! Online for better comprehension and to use an objective and quantitative method.

## Materials and methods

This is a prospective clinical study of one cohort. We surveyed female patients from the Pontificia Universidad Catolica de Chile, Cancer Centre, who had received chemotherapy for early BC and had completed chemotherapy at least three months before the interview. Patients were excluded if they had metastatic BC at the time of interview or did not receive chemotherapy. The patients were interviewed between January 2004 and April 2012. The protocol was approved by the local ethics committee, and all patients signed informed consent.

### Survey

Details from the medical history, BC features, and treatment received were obtained from medical records. At the interview, relevant demographic data were updated (age, date of diagnosis, marital status, number of children, and level of education). Then, a single treatment decision question was asked, offering several alternatives:

‘If you were asked to evaluate the need to receive chemotherapy treatment for BC, knowing your previous experience, what survival benefit makes chemotherapy worthwhile?’

To make sure that the patient understood the concept of survival benefit, we showed her graphics obtained from Adjuvant! Online, displaying several hypothetical scenarios. These cases have different risks and therefore different magnitudes of benefit in overall survival. The options were discrete and incremental, ranging from 1% to 20%.

The poll was conducted by any of the two main investigators (Cesar Sanchez and Francisco Acevedo) using a standardised form and in the context of a spontaneous outpatient visit. The duration of the interview varied from 15 to 20 min.

## Statistical analysis

We analysed the data to assess the relationship between demographic factors, age at diagnosis, type of therapy received, number of children, and level of education, and quantitative estimates of treatment benefit elected. Preferences are presented descriptively with figures and frequency tables.

Associations between characteristics and preferences of patients were assessed with the *X*^2^ test. Because of the low number of patients recruited and to simplify analyses, we decided to dichotomise some variables (e.g., married and de facto: with partner; single, separated, and divorced: without a partner). Logistic regression was used to explore associations between dichotomised dependent and independent variables. A comparison between mean of ages regarding preferences was done with *t*-score for independent variables.

All of the statistical analyses were performed using the program IBM SPSS version 21.

## Results

We interviewed 101 patients, all of whom agreed to participate in the study and signed the consent form. The main characteristics of the participants are shown in Table 1. The average age was 55.9 (±10.2) (median: 57 years and range: 29–76), more than half had lymph node involvement at the time of diagnosis, 20% of patients were stage I, and 41% received a combination of anthracyclines and taxanes as a systemic treatment.

The median of time from last chemotherapy to interview was 19 months (range 3–175). In regard to the level of education, 40% of patients had advanced degrees, most of them were married or de facto, and 17 patients reported that they did not have any children.

**Figure 1:**
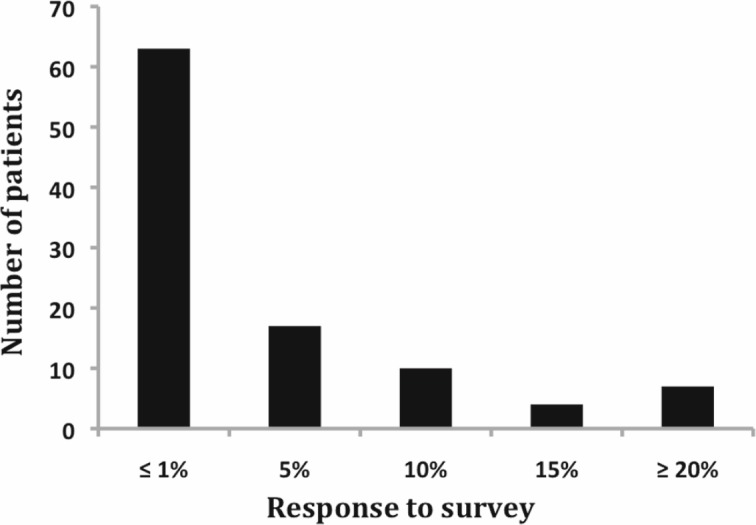
Response to adjuvant survey. Notice that more than 60% of patients decided that chemotherapy was worthwhile receiving for an estimated benefit of 1% or less.

Regarding patient preferences, 63% of respondents decided that chemotherapy was worthwhile to receive for an estimated benefit of 1% or less (Figure 1). On the contrary, 20% of patients judged that more than 10% benefit was necessary to opt for chemotherapy.

Patients who responded 1% or less were four year younger than patients who require larger benefits, although this difference did not reach statistical significance (54.5 [±10.5] versus 58.1 [±9.4] years, *p* = 0.08). In a univariate analysis, only nodal status (*p* = 0.022) and time from end of chemotherapy to survey (*p* = 0.049) were significantly associated with patient’s preferences. We also found a non-significant trend in age and level of education (Table 2).

In the multivariate analysis, being younger (*p* = 0. 02; or 0.9 [0.86–0.98]) and not having a university education (*p* = 0.018; or 5.2 [1.3–20.7]) were significantly associated with requiring 1% or less benefit to consider chemotherapy worthwhile.

## Discussion

This is the first report, to our knowledge, evaluating the perception of Latino patients regarding the benefit of chemotherapy in early BC, especially when they are asked in their own language. Interestingly, most of the patients, as described previously in the Caucasian population, needed a small survival benefit to accept treatment with chemotherapy despite the significant side effects and the inconveniences derived from the treatment.

We found that the presence of lymph node metastasis in the patient’s history (*p* = 0.022) and time interval between the end of chemotherapy to the survey (*p* = 0.049) was associated with patients accepting chemotherapy with smaller survival benefit. Regarding the former, the same results have been reported elsewhere [2]. Our interpretation is that patients who perceive themselves as at higher risk of systemic disease are more likely to accept chemotherapy [6]. Regarding the time interval from chemotherapy, the group that recently finished chemotherapy accepted a smaller survival benefit than the group who was surveyed one year after the completion of treatment. We propose that patients who are just ending treatment are more susceptible to a condition known as ‘cognitive dissonance’ (see below) so they would require a lesser benefit to opt for chemotherapy. Nevertheless, neither of these findings was able to predict response in the multivariate analysis.

On a multivariate analysis, age (*p* = 0.02) and level of education (*p* = 0.018) were the only independent factors associated with the degree of survival benefit. The few statistically significant predictive factors found in our study may be due to a small sample of patients and the low prevalence of the tested characteristics.

Other studies have shown that treatment preferences may also be affected by other demographic variables, such as living with others, being a parent or having dependent children [7]. There is a high agreement between patients and their partners in the benefit considered being worthwhile, which seems to be enhanced in a worse prognosis scenario [8]. Interestingly, in the same paper, having dependent children was associated with partners requiring higher benefits [8].

Although most of the patients with BC prefer to make decisions together with their doctor [9], many physicians remain reluctant to provide patients with detailed information of their treatment and prognosis [5, 10]. Although patients with cancer have to be assessed in a multidisciplinary manner, the uncoordinated evaluation of several physicians may influence information delivery and can complicate the communication process [11, 12].

Moreover, patients with cancer tend to overestimate their benefit with treatment and judged smaller benefits to be worthwhile than medical and nursing professionals [5, 13].

Worldwide, there is a growing interest to involve the patient in decision making. Even though there are patients who do not want to make the final decision, most of them want doctors to understand their preferences [14]. A shared-decision model seeks to promote collaboration between patients and their physicians to reach agreement about multiple appropriate treatment options concerning her/his disease. To make a shared-decision model possible, clinicians should explain treatment options in a language accessible to the patient, highlighting options and discussing the pros and cons of those alternatives.

Risk estimation based on numerical measures is complex and difficult to interpret even for doctors [15]. We represented benefit in absolute terms, and although absolute values like the ‘number needed to treat’ have been suggested as the best approach for easier patient interpretation [16], other studies suggest the opposite [17]. It is not easy to be certain that patients understand this concept. Modification of this value, along with the consideration of the probability of being harmed by the treatment, has made some authors propose the use of the ‘Likelihood of Being Helped or Harmed’ as a better accepted method [18].

To facilitate understanding, visual aids or graphics have been reported to enhance and improve communication with cancer patients [11, 19, 20]. The purpose of these tools is to translate the results from the decision analyses or prognosis from evidence-based studies into friendlier formats. Peele *et al* [20] showed in a randomised trial that in patients whose benefits were small, the use of a decision aid helped women to decide whether to receive chemotherapy or not [20].

However, even with the use of visual aids, do they really understand the concept of benefit? Would they accept chemotherapy for no benefit at all? Jansen *et al* [21] reported that 40% of patients would accept the treatment for no benefit and 36% of our patients would consider chemotherapy for less than 1% benefit. Chemotherapy may provide vulnerable BC patients with a feeling that they are doing something active to deal with their disease [22] so the final decision could actually represent a mechanism of protection.

Time is crucial for this system to work. The time we spent to explain patient’s benefits using Adjuvant! graphics was 15–20 min on average. Poor reimbursement for time spent in a long discussion about pros and cons may be unattractive to some oncologists. Guy and Richardson [23] found that the length of time physicians spend with their patients may vary across different characteristics. It is unclear whether this could affect information delivery or not.

The main strength of our report is that we were able to estimate the magnitude of benefit required for Latino patients to consider chemotherapy worthwhile, when asked in their native language and in their own country. Race/ethnicity and culture influence perception and communication with physicians [24]. In the United States, there is evidence suggesting that black patients with BC have a poorer prognosis and survival rates comparing with white patients [25]. This disparity has been noticed, to a lesser extent, in Hispanic patients too [26]. Part of this difference might be explained as due to biologic factors [27, 28], but there are data that show that Latino patients are less likely to report receiving a mammography or to participate in healthcare decisions, with half of the final treatment resolution being determined by their families [29, 30]. Adherence to medical suggestions in this population depends on various factors including a preference to being interviewed in Spanish and physician involvement [31, 32].

Despite communication issues, Hispanics did not show evidence of under treatment in a large, diverse, population-based sample of women diagnosed with BC, regardless of the level of acculturation [33]. On the contrary, low acculturated Hispanics were the most likely to report having received adjuvant chemotherapy than better educated Hispanics or black patients. Could this be attributed to a lack of patient– physician interaction about the pros and cons of treatment because of a language barrier? Low acculturated patients may be more likely to agree to a treatment that they do not fully understand. Although this point was not an objective of our paper, we think that this limitation may be overcome with the use of visual aids.

Our paper has several limitations. This survey was conducted only in patients who had previously received chemotherapy. We decided to restrict our study to this population because of their personal knowledge of the adverse effects of the treatment.

There is also evidence that suggests that vulnerable patients who had experienced situations that are not consistent (dissonance) with their thoughts or beliefs, such as chemotherapy, may remain reluctant to retrospectively visualise other options as valid. This phenomenon known as ‘cognitive dissonance reduction’ is a coping strategy that tries to take the subject to a new balance, minimising regrets about past decisions [34]. One method to overcome the bias is to survey the same patient before and after treatment. In a small report by Jansen *et al* [21], patients who did not receive chemotherapy would accept larger benefits than patients who received the treatment (*p* < 0.01).

An important limitation is that we do not collect data regarding QoL or whether the patients were able to complete treatment or not. This is important for interpretation of the data since it is probable that the patients who tolerated chemotherapy without side effects and completed the planned treatment would accept chemotherapy for a smaller benefit. This is highlighted by the fact that the study was performed on patients presenting to our cancer centre spontaneously. Those are probably the patients who maintained regular medical controls, and the ones who most often had a better experience in relation to the chemotherapy.

The clinical implications of this study are crucial for our daily practice. How much benefit do we need or ‘how low’ can we go when we offer chemotherapy to our patients? How do we integrate these two different appreciations of what is good for them? Clinicians should explain the pros and cons of adjuvant chemotherapy and allow their patients’ views, values, and priorities to influence discussions and decisions about adjuvant chemotherapy.

## Conclusion

Most of the patients in a Latin cohort who had experienced chemotherapy for BC, accepted treatment with a small survival benefit. The results are comparable with those in other publications elsewhere. We emphasise the need for an active discussion of the pros and cons of chemotherapy with BC patients considering adjuvant treatment and that it is essential to take their personal beliefs and opinions into consideration.

## Conflicts of interest

The authors have no conflicts of interest to declare.

## Figures and Tables

**Table 1.** Demographics characteristics of the patients.

	Characteristics	*N* = 101	Percentage (%)
Stage (TNM)	1	20	19.8
	2	48	47.5
	3	25	24.7
	Unknown	8	7.9
Nodal Stage	Positive	55	54.4
	Unknown	8	7.9
Hormonal receptor	Positive	77	76.2
	Negative	23	22.8
	Unknown	1	1.0
Age at interview	≤ 50: 27.7	28	26.7
	51-64: 52.5	53	52.4
	≥ 65: 19.8	20	20.9
Marital status	Married or de facto	65	64.3
	Single	21	20.8
	Separated or divorced	11	10.9
	Widowed	4	3.9
Children	Has Children	85	15.8
	None	16	84.2
Highest level of education	None	2	1.9
	Primary school	17	16.8
	High school	42	41.6
	University	40	39.6
Time (months) since last chemotherapy	3–12	33	32.6
	13–59	34	33.7
	≥ 60	34	33.7
Adjuvant chemotherapy	Anthracyclines (FAC–AC)	53	52.4
	Anthracyclines and taxanes (AC-T and FEC-D)	42	41.6
	Others (taxanes, CMF and EP)	5	4.9
	Unknown	1	1

**Table 2.** Response based on subgroups.

	Characteristics	≤ 1% *N* = 63	> 5% *N* = 38	Significance (*p*)
Age	*X*	54.5% (±10.5%)	58.1% (±9.4%)	0.088
Stage (TNM)	1	13/57 (22.8%)	7/36 (19.4%)	0.12
	2	25/57 (38.6%)	23/36 (63.9%)	
	3	19/57 (33.3%)	6/36 (16.7%)	
Nodal Stage	Negative	18/57 (31.6%)	20/36 (55.6%)	0.022^*^
	Positive	39/57 (68.4%)	16/36 (44.4%)	
Hormonal receptor	Positive	45/62 (72.6%)	32/38 (84.2%)	0.22
	Negative	17/62 (27.4%)	6/38 (15.8%)	
Marital status	With partner	40/63 (63.5%)	25/38 (65.8%)	0.8
	Without a partner	23/63 (36.5%)	13/38 (34.2%)	
Children	None	9/63 (14.3%)	7/38 (18.4%)	0.5
	Has children	54/63 (85.7%)	31/38 (81.6%)	
Highest level of education	None	42/63 (66.7%)	19/38 (50.0%)	0.09
	University	21/63 (33.3%)	19/38 (50.0%)	
Time (months) since last chemotherapy	3–12	25/63 (52.8%)	8/38 (21.1%)	0.049^*^
	13–59	16/63 (25.4%)	18/38 (47.4%)	
	≥ 60	22/63 (34.5%)	12/38 (31.6%)	
Adjuvant chemotherapy	Anthracyclines and taxanes	29/59 (49.1%)	13/38 (34.2%)	0.14
	Anthracyclines or taxanes	30/59 (50.9%)	25/38 (65.8%)	

* Statistically significant difference (*p* < 0.05).
